# Engineering strategies and decision frameworks for virus-like particle-based vaccines against infectious diseases

**DOI:** 10.3389/fmicb.2026.1795711

**Published:** 2026-04-23

**Authors:** YiYan Liu, Yanan Zhang, Lijuan Liang, Han Zhang, Tianjiao Zhang, Xifeng Rong, JiYing Tan, Youjun Mi

**Affiliations:** 1 Department of Pathophysiology, School of Basic Medical Sciences, Lanzhou University, Lanzhou, China; 2 The First Hospital of Lanzhou University, Lanzhou, China; 3 The First Clinical Medical School of Gansu University of Chinese Medicine, Lanzhou, China; 4 Cuiying Honors College of Lanzhou University, Lanzhou, China; 5 Department of Immunology, School of Basic Medicine Sciences, Lanzhou University, Lanzhou, China

**Keywords:** antigen display, infectious diseases, nucleic acid delivery, vaccines, virus-like particles (VLPs)

## Abstract

Virus-like particles (VLPs) have emerged as a versatile and clinically validated platform for developing safe, effective vaccines against infectious diseases. However, the expanding toolkit of VLP engineering strategies–spanning genetic fusion, modular conjugation, and nucleic acid encapsulation–creates a critical need for a rational selection framework to match technological strengths with specific vaccine objectives. This review addresses this gap by constructing a comparative decision-making framework centered on four core engineering dimensions: cargo flexibility, loading specificity, functional efficiency, and manufacturability. We systematically juxtapose two principal technology streams: (1) the display of protein antigens (through genetic, chemical, and bio-conjugation) and (2) the encapsulation of nucleic acid cargo (via physical, electrostatic, and programmable packaging mechanisms), evaluating each within this unified framework. This technological dissection is directly linked to the development landscape of VLP-based vaccines against major pathogens–including HBV, HPV, malaria, influenza, and SARS-CoV-2–illustrating how strategic choices at the engineering level fundamentally underpin immunogenic potency and translational success. By sequentially considering immunological objectives, antigen compatibility, surface display modality, interior cargo integration, and manufacturing constraints, this framework facilitates rational, stepwise VLP vaccine design. Looking forward, we discuss emerging trends toward modular and computationally guided platforms for antigen placement and scaffold design. By integrating a structured technology assessment with translational insights, this review aims to provide a practical roadmap for the rational design and accelerated development of next-generation, broadly protective VLP-based vaccines.

## Graphical Abstract

**Figure:**
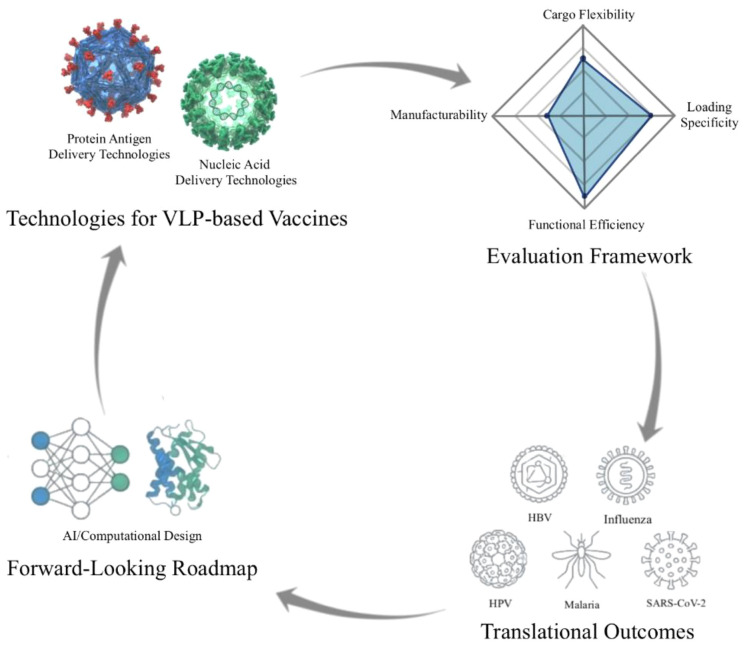
A unified analytical framework for rational VLP platform matching. The roadmap illustrates the trade-off logic between two principal technology streams—protein antigen display and nucleic acid encapsulation—evaluated across four core engineering dimensions: cargo flexibility, loading specificity, functional efficiency, and manufacturability. By integrating these engineering considerations with translational outcomes and AI-driven computational design, this cycle provides a structured pathway for the development of next-generation VLP-based vaccines.

## 1 Introduction

Virus-like particles (VLPs) are nanoscale assemblies formed through the self-organization of viral structural proteins. By preserving the native conformational epitopes of authentic virions while eliminating infectious genetic material, VLPs elicit potent humoral and cellular immune responses without the risks associated with replication. This unique combination of immunogenicity and safety has established VLPs as a cornerstone platform for next-generation vaccine development (Bachmann et al., 2025; Kheirvari et al., 2023). Since the first identification of non-infectious, virus-like structures in human serum in 1968 (Bayer et al., 1968), VLPs have evolved from biological curiosities into highly adaptable scaffolds widely deployed across prophylactic, therapeutic, and molecular-delivery applications.

The maturation of VLP engineering over the past decade has dramatically expanded their functional scope, reflecting a shift from largely empirical assembly toward increasingly programmable molecular architectures. Advances such as SpyTag–SpyCatcher covalent coupling (Brune et al., 2016; Zakeri et al., 2012), sortase-mediated ligation (Patterson et al., 2017), and aptamer- or RNA-guided cargo encapsulation (Fiedler et al., 2010) provide precise spatial, stoichiometric, and sequence-level control over how antigens or functional nucleic acids are integrated into VLP architectures. These tools address historical limitations including low cargo density, limited site specificity, and susceptibility to structural instability. However, each strategy operates under distinct engineering constraints that shape its translational suitability. For example, SpyTag–SpyCatcher conjugation offers high reaction robustness but may be restricted by expression yield, whereas programmable RNA-guided encapsulation affords superior specificity at the expense of sequence-design complexity. The incorporation of environmentally responsive elements and rationally designed protein scaffolds further extends the versatility of engineered VLPs, enabling applications spanning prophylactic vaccination, targeted gene delivery, and immunotherapeutic modulation.

Clinically, VLPs have demonstrated substantial translational value. Approved vaccines against hepatitis B virus (HBV), human papillomavirus (HPV), and hepatitis E virus (HEV) exemplify the capacity of VLP-based platforms to induce durable and protective immunity (Diaz-Mitoma et al., 2021; Joura et al., 2015; Zhu et al., 2010). Parallel preclinical and clinical efforts targeting malaria, influenza, SARS-CoV-2, and human immunodeficiency virus (HIV) continue to refine VLP architectures for broader antigenic coverage, improved manufacturability, and enhanced stability under real-world deployment conditions (Agnandji et al., 2012; Collins et al., 2017; Kanekiyo and Graham, 2021; Olotu et al., 2011; Rosengarten et al., 2022; Walls et al., 2020; Zhang et al., 2015). These developments reinforce an emerging need for systematic criteria to guide the rational selection of VLP formats tailored to distinct pathogen targets and immunological objectives.

As the diversity of VLP platforms continues to expand, vaccine development is increasingly moving toward rational platform matching. While structural differences in geometry, scale, and assembly pathways define important boundary conditions for platform selection, the practical challenge lies in coordinating antigen density, cargo control, and manufacturing feasibility within these structural frameworks. Building on this landscape, this review integrates contemporary VLP engineering strategies into a unified analytical framework. Rather than providing an exhaustive classification of structural variants, we focus on comparing the adaptability and trade-off logic of different engineering streams in real-world development scenarios. We compare two principal technology streams–(1) protein antigen display via genetic fusion, chemical conjugation, and bio-orthogonal modular assembly, and (2) nucleic acid encapsulation through physical, electrostatic, and programmable mechanisms–and evaluate them against four core engineering dimensions: cargo flexibility (types and sizes of antigens or nucleic acids accommodated), loading specificity (binding strength and achievable density), functional efficiency (site-selective orientation and immunological potency), and manufacturability (production complexity and host system requirements). By linking these engineering considerations to ongoing clinical and preclinical programs, we outline a structured analytical framework to inform rational design and platform selection in next-generation VLP-based vaccine development.

## 2 Engineering streams for VLP customization: from surface display to interior loading

The rapidly diversifying landscape of VLP customization strategies can be conceptualized as two complementary engineering streams: (1) exterior protein antigen display, and (2) interior nucleic acid encapsulation. Each stream encompasses distinct modalities that differ in their biophysical principles, stoichiometric precision, and translational robustness. To facilitate a systematic comparison, we evaluate these modalities through a standardized four-dimensional framework comprising cargo flexibility, loading specificity, functional efficiency, and manufacturability. This framework provides a rigorous basis for assessing how each strategy navigates the inherent structural constraints of the VLP scaffold, the metabolic limits of production hosts, and the stringent requirements of clinical-grade vaccine development (Tables 1, 2).

**TABLE 1:** Exterior display strategies: a decision matrix.

Engineering dimension	Genetic fusion (section 2.1.1)	Modular strategies (section 2.1.2)	Chemical conjugation (section 2.1.3)
Cargo flexibility	Restricted: linear peptides < 50 aa; sensitive to pI/hydrophobicity	Broad: globular proteins > 50 kDa; tolerates PTMs	Maximum: proteins, glycans, lipids, synthetic molecules
Loading specificity	Absolute: genetically encoded 1:1 stoichiometry	Programmed: site-specific via enzymatic/affinity tags	Stochastic: random distribution on lysine/cysteine residues
Homogeneity	Highest: fixed geometry; periodic nanoscale array	High: defined orientation; high occupancy (>90%)	Variable: polydisperse display; orientation-agnostic
Manufacturability	Streamlined: single-host expression; lower COGs	Complex: multi-component production; *in vitro* coupling required	Scalable: rapid post-assembly functionalization
Critical bottleneck	Capsid misfolding: risk of off-pathway aggregation or inclusion bodies	Process control: balancing molar ratios and enzymatic activity	Batch consistency: site-occupancy validation challenging
Strategic recommendation	Best for well-defined, short linear epitopes requiring maximum density	Best for high-value recombinant antigens (e.g., RBD, trimers)	Best for non-proteinaceous cargos or rapid prototyping
Representative examples	HBc VLP – CS B repeat (Yan et al., 2015); HBsAg VLP – HPV E7 (Kim and Kim, 2017)	SpyCatcher-mi3 – SARS-CoV-2 RBD (Tan et al., 2021); AP205 – *Shigella oligosaccharides* (Hills and Howarth, 2022)	P22 VLP – *Burkholderia* Hcp1 (Khakhum et al., 2024); HepBc SS1 – *Chlamydia* antigens (Chan, 2014)

**TABLE 2:** Interior loading strategies: an encapsulation framework.

Engineering dimension	Disassembly–reassembly (section 2.2.1)	Charge-mediated (section 2.2.2)	Sequence-guided (section 2.2.3)	Structure-guided (section 2.2.4)
Cargo flexibility	Maximum: nucleic acids (any length), small molecules, enzymes	Broad: anionic mRNA, siRNA, pDNA; sensitive to length/stiffness	Defined: target RNA fused to specific viral PS motifs	Programmable: mRNA, circRNA, sgRNA with aptamer tags
Loading specificity	Stochastic: bulk physical entrapment; lack of molecular cues	Bulk-driven: electrostatic sequestration; low selectivity	Programmed: sequence-specific PS–capsid recognition	Absolute: orthogonal “lock-and-key” aptamer–ABP pairs
Functional efficiency	Lowest: significant fluctuations; mix of empty/full particles	Variable: high polydispersity; risk of surface adsorption	High: defined stoichiometry; genome-mimetic occupancy	Highest: precise molar control; standardized 1:1 recruitment
Manufacturability	Technically demanding: precise titration of ionic/redox gradients	Streamlined: simple mixing under mild buffer conditions	Complex: coupled biosynthetic regime; *in vivo* co-expression	High overhead: multi-component balance; fragile co-assembly equilibria
Critical bottleneck	Thermodynamic limit: steric exclusion for cargos > 3 kb; aggregation risk	Physicochemical stability: dissociation at physiological salt (∼150 mM)	Expression kinetics: balancing host metabolic load with assembly rate	Batch consistency: sensitivity to protein–RNA folding and molar ratios
Strategic recommendation	Best for exploratory studies and high-volume, non-specific payloads	Best for rapid screening and simple, small anionic nucleic acid cargos	Best for sustained intracellular expression where off-target packaging is minimized	Best for high-precision therapeutics requiring modularity and maximal selectivity
Representative examples	HPV L1 – RSV antigens (Graham et al., 2010); Murine polyomavirus – pDNA (Kanekiyo and Graham, 2021)	HBc (ARD) – pDNA (Warner and Frietze, 2021); Qβ VLP – vaccine antigens (Kim et al., 2026)	PEG10 – CRISPR-Cas9 (Segel et al., 2021); HIV PS – neoantigen mRNA (Haddrick et al., 1996)	MS2 (MCP) – PSA mRNA (Lyu and Lu, 2022); PP7 – circRNA (Shirbaghaee and Bolhassani, 2016)

### 2.1 Extra-VLP protein antigen display technologies

**FIGURE 1:**
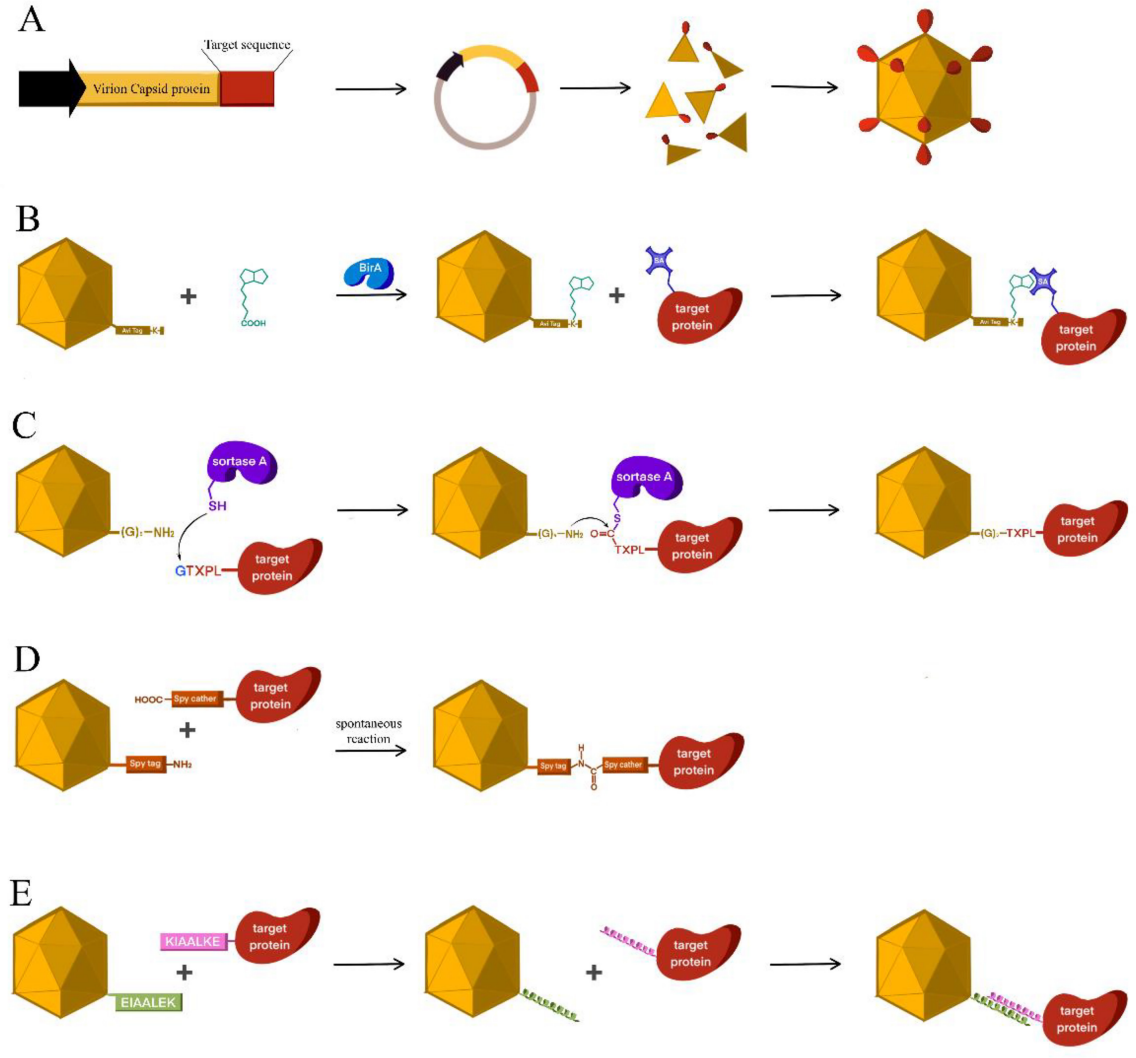
Biological and modular modalities for extra-VLP antigen display. **(A)** Genetic fusion: a recombinant, gene-level insertion strategy where exogenous antigens (full-length or epitopes) are integrated into structural proteins at permissive termini or surface-exposed loops, enabling co-assembly during particle formation. **(B)** Streptavidin–biotin interaction: a high-affinity, non-covalent capture modality where biotinylated VLP scaffolds–generated via chemical or enzymatic modification–bind streptavidin-tagged antigens through one of the strongest known non-covalent interactions. **(C)** Sortase-mediated ligation: a site-specific enzymatic conjugation approach in which Sortase A catalyzes transpeptidation between an LPETG-tagged antigen and N-terminal oligoglycine motifs on the VLP, forming a stable covalent peptide linkage. **(D)** SpyTag–SpyCatcher system: a modular covalent coupling strategy based on spontaneous isopeptide bond formation between SpyTag and SpyCatcher components, supporting irreversible antigen attachment under mild conditions. **(E)** Coiled-coil assembly: a non-covalent display strategy utilizing the selective heterodimerization of complementary α-helical coiled-coil pairs to achieve directional and affinity-driven antigen loading.

**FIGURE 2:**
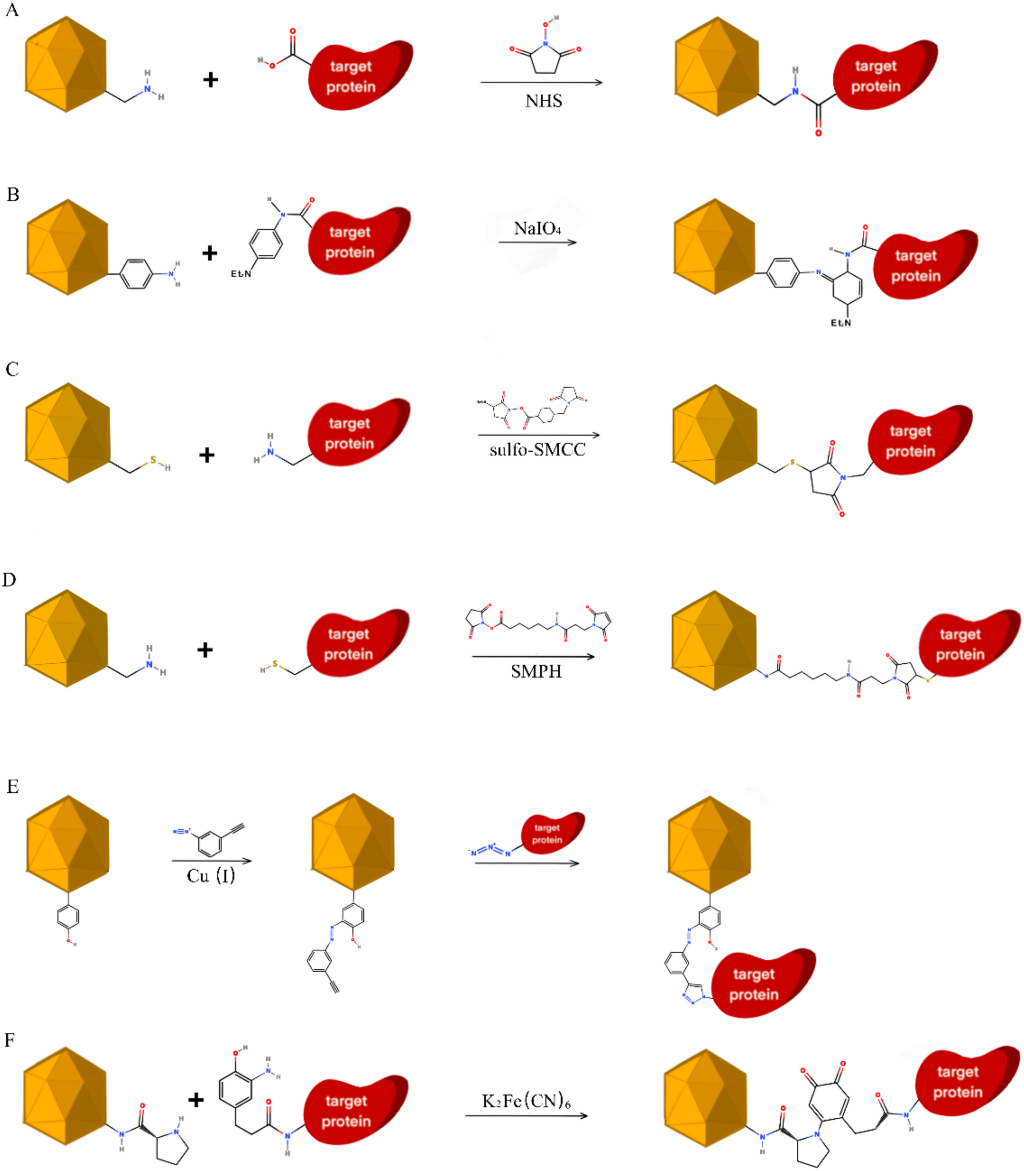
Chemical bioconjugation strategies for extra-VLP antigen display. **(A)** Carbodiimide-mediated coupling: EDC/Sulfo-NHS activation of carboxyl groups to form reactive NHS esters, facilitating amide bond formation with primary amines on the VLP or antigen. **(B)** Oxidative aniline–aromatic coupling: NaIO_4_-mediated oxidative reaction between aniline-modified VLP surfaces and aromatic amine-containing moieties on the antigen. **(C)** Maleimide–sulfhydryl ligation: site-specific conjugation between maleimide-functionalized antigens and VLP sulfhydryl groups within a defined pH range (6.5–7.5). **(D)** Heterobifunctional crosslinking: bridging of VLP-surface amines and antigen-surface thiols via sequential NHS-ester and maleimide chemistry using linkers [e.g., SMPH, SM(PEG)_4_]. **(E)** Tyrosine-targeted click chemistry: modification of VLP tyrosine residues with diazonium salts for alkyne introduction, followed by copper-catalyzed azide–alkyne cycloaddition (CuAAC). **(F)** N-terminal proline ligation: site-specific covalent conjugation via the reaction of N-terminal proline imino groups with oxidized phenolic intermediates to form stable conjugates.

Irrespective of the specific engineering stream, the governing design principle remains consistent: selecting a delivery modality whose intrinsic mechanistic attributes are precisely aligned with defined immunological objectives and manufacturing constraints. This section focuses on exterior protein antigen display strategies, systematically evaluating the principal methodologies through the four-dimensional decision framework introduced above. To facilitate this evaluation, we categorize surface engineering into two distinct streams: biological and modular modalities (Figure 1), which rely on genetic and protein engineering, and chemical bioconjugation strategies (Figure 2), which utilize site-specific covalent chemistry. We begin with genetic fusion, the foundational and most extensively implemented modality for VLP surface engineering.

#### 2.1.1 Genetic fusion: balancing antigen presentation with structural integrity

Genetic fusion is an integrated strategy that uses genetic engineering to fuse an antigen-coding sequence directly with a viral structural protein gene, enabling co-expression and co-assembly of chimeric VLPs (Figure 1A). By incorporating the antigen as an intrinsic structural element of the particle, this approach provides precise spatial control over antigen display, high stoichiometric uniformity, and superior particulate homogeneity–advantages that are difficult to achieve with post-assembly conjugation or non-covalent methods (Grgacic and Anderson, 2006). Production typically relies on a single-expression system, most often in *E. coli* or yeast, which simplifies manufacturing and scale-up but imposes strict constraints on the size and folding of the antigen (Lua et al., 2014).

From a biophysical perspective, genetic fusion represents a “coupled assembly system” where the antigen’s presence directly influences the capsid’s self-assembly energy landscape. The feasibility of this approach is inherently governed by the spatial and geometric symmetry of the VLP scaffold (Bachmann and Jennings, 2010). In highly ordered platforms, such as icosahedral capsids, the fixed arrangement of subunits creates significant steric hindrance; attempting to fuse large or structurally rigid domains can disrupt the localized subunit interactions and lead to off-pathway kinetic traps or amorphous aggregates (Katen and Zlotnick, 2009). Experimental insertion mapping studies in MS2 and HBc platforms demonstrate that structured surface loops typically tolerate short peptides (approximately 20–40 amino acids), whereas larger insertions frequently impair particle assembly (Peabody et al., 2008; Pumpens and Grens, 2001). Sequences rich in positively charged, hydrophobic, or β-strand-prone residues may further induce misfolding, exceeding the entropic and structural boundary conditions required for ordered assembly (Janssens et al., 2010).

Despite these constraints, genetic fusion delivers exceptional product specificity and homogeneity, as antigens are displayed at predefined geometric positions with fixed stoichiometry. This enables dense, repetitive antigen arrays that potently activate B cells through multivalent B-cell receptor (BCR) cross-linking (Grgacic and Anderson, 2006). Advanced protein engineering can partially mitigate size limitations; for example, single-chain dimer designs (e.g., in MS2 bacteriophage) link the N- and C-termini of coat protein dimers into a single open reading frame, effectively reducing the entropic penalty during assembly (Frietze et al., 2016) and creating additional insertion-permissive sites such as the AB-loop (Peabody et al., 2008).

Platform-specific examples illustrate the versatility and limits of this approach. Hepatitis B core (HBc) VLPs are among the most insertion-tolerant carriers due to the inherent flexibility of their spike structures (Pumpens and Grens, 2001), allowing epitopes to be displayed at the N-terminus, C-terminus, or the major immunodominant region (MIR). For example, the vaccine candidate MalariVax was produced by inserting CSP T-epitopes at the C-terminus of truncated HBc and the T1/B repeat epitopes into the MIR loop (Nardin et al., 2004). Experimental studies have also demonstrated that HBsAg VLPs can accommodate heterologous epitopes such as HPV E7 (19 aa) (Pumpens et al., 2002), and HPV L1 VLPs can successfully assemble with up to 60 amino acids of HPV-16 E7 fused to the L1 C-terminus (Monroy-García et al., 2011).

From a manufacturing standpoint, although genetic fusion utilizes a simplified single-expression system, fusion constructs frequently require extensive, antigen-specific optimization because large or structurally complex antigens often express as insoluble aggregates or inclusion bodies, as exemplified by HBc fusions with SARS-CoV-2 RBD in which only low yields of soluble VLP were obtained without scaffold modification (Petrovskis et al., 2024). The inherent incompatibility between certain antigen folds and capsid architectures can significantly reduce assembly efficiency, especially when the antigen exceeds the physical capacity of the display site (Gupta et al., 2023; Tariq et al., 2022). Ultimately, genetic fusion is a strategic choice prioritizing structural precision. When antigen complexity threatens capsid stability, a transition to modular “plug-and-display” strategies–which decouple scaffold production from antigen engineering–becomes the necessary engineering pivot (Brune and Howarth, 2018).

##### 2.1.1.1 Decision triggers for genetic fusion selection

Genetic fusion is preferentially selected when antigen size, topology, and folding requirements are fully compatible with scaffold assembly constraints. It is particularly suited for short linear epitopes (≤40–50 aa) or compact protein domains that can be genetically inserted without disrupting capsid symmetry, self-assembly kinetics, or particle stability.

This strategy is optimal when strict stoichiometric control, uniform antigen occupancy, and fixed geometric spacing are essential for immunogenic efficacy–such as in applications relying on repetitive epitope arrays for efficient B cell receptor cross-linking.

Strategic pivot to modular or post-assembly approaches is warranted when the antigen exceeds structural tolerance thresholds (e.g., >50 kDa), requires independent eukaryotic folding or glycosylation, or when fusion perturbs particle assembly, reduces yield, or induces structural heterogeneity.

#### 2.1.2 Modular strategies: interaction-programmed “plug-and-display” architectures

Specific interaction–mediated bioconjugation represents a modular approach for functionalizing VLPs, in which pre-assembled antigens are attached to VLP scaffolds via highly selective molecular interactions. From a biophysical perspective, this strategy operates in a “thermodynamically decoupled” regime, where antigen folding and capsid assembly are optimized independently. By protecting the intrinsic assembly energy landscape of the VLP, this approach prevents perturbations to local thermodynamic minima that can trap subunits in off-pathway, amorphous aggregates–a frequent failure mode in genetic fusions on highly symmetric icosahedral VLPs (Figure 1A; Guo et al., 2024; Zlotnick, 2005). Practically, this decoupling allows for two separate production streams, enabling independent optimization of folding and post-translational modifications, though it necessitates balancing antigen size with manufacturing complexity and production economics (Balbierer et al., 2025; Park et al., 2023; Sun et al., 2024a,b).

##### 2.1.2.1 Affinity-driven modularity: streptavidin–biotin as a universal adapter

The streptavidin–biotin system leverages ultrahigh affinity (dissociation constant, Kd ≈ 4 × 10^14^ M) to enable non-covalent yet exceptionally stable antigen display (Figure 1B; Dundas et al., 2013; Howarth et al., 2006). In this architecture, the VLP scaffold functions as a universal docking hub, where inter-epitope spacing is governed by the intrinsic lattice periodicity of the capsid. This preserves the dense, repetitive nanoscale geometry required for multivalent BCR clustering without perturbing the underlying capsid structure (Halfmann et al., 2022; Thrane et al., 2015). Typically, VLPs are first biotinylated–via chemical means or site-specific enzymatic labeling (e.g., BirA ligase)–and subsequently used to capture streptavidin-conjugated antigens (Bárcena and Blanco, 2013; Chackerian et al., 2001; Chen et al., 2005; Fairhead and Howarth, 2015; Schiller and Lowy, 2001; Schwarz et al., 2017).

This approach imposes fewer structural constraints than genetic fusion strategies and can accommodate large protein antigens (Brune et al., 2016; Lampinen et al., 2021). A major biochemical strength is that biotin binding markedly increases streptavidin’s thermal stability (Tm: 73 °C → 112 °C), enhancing formulation robustness (González et al., 1999). Additionally, its lower isoelectric point and lack of glycosylation relative to avidin reduce non-specific interactions (Balzer and Whitehurst, 2023). To manage product heterogeneity, monovalent streptavidin variants are often employed to prevent the inter-particle cross-linking inherent to tetravalent wild-type proteins (Howarth et al., 2006; Wu and Wong, 2005). The effectiveness of this adapter is demonstrated by MS2 VLPs displaying SARS-CoV-2 Spike proteins (Setyo Utomo et al., 2023) and Avi-tagged HPV L1 VLPs docking VAR2CSA malaria antigens (Thrane et al., 2015), both of which maintained high epitope accessibility and structural integrity.

##### 2.1.2.2 Enzymatic ligation: Sortase A–mediated covalent precision

Sortase A–mediated transpeptidation offers a site-specific covalent bond between an LPETG-tagged antigen and an N-terminal oligoglycine motif on the VLP surface (Figure 1C; Brune and Howarth, 2018; Frietze et al., 2016; Obeng et al., 2023). This enzymatic strategy provides greater positional control compared to non-covalent or stochastic conjugation approaches. Under mild, near-neutral conditions, reported coupling efficiencies reach ∼90%, enabling the assembly of uniform and immunogenic particles (Olivera-Ugarte et al., 2022; Thérien et al., 2017).

From a manufacturing standpoint, this site-specific precision enhances product homogeneity and improves batch-to-batch consistency (Morgan et al., 2022; Obeng et al., 2023). However, achieving high ligation efficiency typically necessitates a molar excess of substrate to drive the reaction toward completion, frequently reported in the 5–10-fold range in optimized systems (Morgan et al., 2022; Tang et al., 2016). Furthermore, the requirement for stringent downstream purification to ensure complete enzyme removal adds to the overall process complexity and remains a primary technical constraint for large-scale production (Morgan et al., 2022; Obeng et al., 2023). Despite these scalability trade-offs, Sortase A has successfully enabled the dense display of SARS-CoV-2 RBD on PapMV VLPs (Olivera-Ugarte et al., 2022) and EV71 epitopes on HBc particles (Tang et al., 2016), eliciting robust humoral responses.

##### 2.1.2.3 Covalent self-assembly: SpyTag–SpyCatcher isopeptide bond formation

The SpyTag–SpyCatcher system extends modular design to a regime of genetically programmed irreversibility, where an intramolecular isopeptide bond forms autonomously upon interaction (Figure 1D; Bruun et al., 2018; Pechsrichuang et al., 2021). This architecture combines the thermodynamic independence of modular production with the covalent permanence of genetic fusion. In optimized scaffold systems such as SpyCatcher003-mi3, near-quantitative surface occupancy has been achieved with minimal molar excess (≈ 1.5×) (Bruun et al., 2018; Tan et al., 2021). The resulting conjugates exhibit enhanced thermal and pH stability relative to non-covalent assemblies, often preserving structural integrity under conditions that would denature individual components (Brune et al., 2016; Bruun et al., 2018).

Post-assembly conjugation preserves capsid folding, allowing the display of structurally demanding antigens (Liu et al., 2022). While the relatively large docking domains (∼116 aa) may impose steric boundary conditions, these are effectively managed through structure-guided linker engineering or precise modulation of surface display density (Brune and Howarth, 2018; Hills and Howarth, 2022). Consequently, the system’s robustness has been validated across a diverse range of scaffolds, including SpyCatcher003-mi3 VLPs for SARS-CoV-2 RBD (Tan et al., 2021), SpyCatcher-60 scaffolds for multi-antigen MPXV presentation (Belghith et al., 2025), and AP205/Qβ VLPs for *Shigella oligosaccharides* (Hills and Howarth, 2022), as well as platforms spanning HBsAg, Norovirus, and Porcine Circovirus VLPs (Boonyakida et al., 2024; Heinimäki et al., 2022; King et al., 2024; Lampinen et al., 2023; Liu et al., 2022; Marini et al., 2019; Peyret et al., 2020).

##### 2.1.2.4 Stimuli-responsive interfaces: coiled-coils–the smart interface

Coiled-coil motifs represent a specialized class of programmable interfaces where complementary α-helical peptides enable high-affinity (nanomolar Kd) yet reversible association (Figure 1E; Jha et al., 2025; Utterström et al., 2021). Unlike permanent covalent bonds, these “smart” interfaces prioritize temporal and spatial control, allowing for pH-dependent dissociation in response to endosomal acidification (pH < 6.0), a feature utilized to modulate intracellular processing (Utterström et al., 2021).

In helical architectures like TMV, coiled-coils permit repetitive axial insertions that maintain high-density molecular patterns along the longitudinal axis (Balke and Zeltins, 2019; Hewagama et al., 2023). While this modularity offers a uniquely tunable design space, it requires balancing responsive dissociation against the risk of premature release *in vivo* and the potential immunogenicity of the engineered domains (Aumiller et al., 2018; Fletcher et al., 2013; Jha et al., 2025). This strategy has been successfully deployed in vaccine candidates for HIV, influenza, malaria (P27A/P27 on SAPN platforms), and *Streptococcus pneumoniae*, showcasing its utility for vaccines that benefit from dynamically regulated antigen presentation (Babapoor et al., 2011; Boato et al., 2007; El Bissati et al., 2014; Hewagama et al., 2023; Karch et al., 2017; Ogrina et al., 2023; Pimentel et al., 2009; Tamborrini et al., 2015).

##### 2.1.2.5 Decision triggers for modular “plug-and-display” selection

Modular strategies are preferentially selected when antigen structural independence must be preserved–particularly for conformational proteins (>50 kDa), glycosylated antigens requiring eukaryotic expression, or epitopes whose immunogenicity depends on correct tertiary folding.

This regime is advantageous when antigen orientation, high-density display, or multicomponent co-presentation must be independently optimized without perturbing scaffold assembly.

Strategic pivot is warranted if the antigen is a short linear peptide (≤40 aa) compatible with direct genetic insertion, or when dual-stream production and downstream conjugation steps impose unacceptable manufacturing complexity relative to fusion-based systems.

#### 2.1.3 Platform strategy: chemical conjugation–maximum decoupling and cargo flexibility

Chemical conjugation represents the most decoupled form of VLP engineering, where the biogenesis of the scaffold is fully separated from antigen functionalization. Unlike genetic fusion (Section “2.1.1 Genetic fusion: balancing antigen presentation with structural integrity”), which operates within a coupled assembly energy landscape, chemical strategies exploit covalent chemistry to attach antigens to reactive side chains on pre-assembled VLPs. This approach maintains the structural integrity and natural folding environment of the capsid, effectively avoiding the assembly interference and non-specific aggregation frequently associated with co-translational folding (Akache et al., 2016; Brune and Howarth, 2018; Chackerian, 2007). This decoupling enables the use of diverse expression systems–including *E. coli*, yeast, insect, and mammalian cells–allowing independent, parallel optimization of capsid folding and antigen post-translational modifications (Fuenmayor et al., 2017).

The primary strength of this approach lies in its very broad cargo compatibility, encompassing full-length proteins, glycans, nucleic acids, and synthetic molecules that resist direct genetic encoding (Akache et al., 2016; Brune and Howarth, 2018; Chackerian, 2007). However, maximal versatility is intrinsically linked to product heterogeneity. Targeting multiple native residues produces conjugates with variable attachment sites, stochastic orientations, and non-uniform stoichiometries, which can compromise epitope accessibility and batch-to-batch reproducibility (Bachmann and Jennings, 2010; Bárcena and Blanco, 2013; Vaidya and Solomon, 2022). From an immunological perspective, this structural randomness may suboptimize the precise nanoscale periodicity required for potent BCR cross-linking. Empirical studies using DNA-origami and synthetic nanoparticles (Brooks et al., 2023; Veneziano et al., 2020) demonstrate that an epitope spacing of 10–30 nm is a critical determinant for robust BCR signaling, a parameter that is inherently difficult to standardize in stochastic chemical conjugation systems. Consequently, chemical conjugation serves as a strategic fallback platform for antigens unsuitable for modular or genetic strategies, where managing structural diversity is a necessary compromise for achieving broad functionalization.

##### 2.1.3.1 The chemical toolbox: foundational amine- and thiol-based bioconjugation

Carbodiimide-mediated coupling (EDC/NHS): carbodiimide-mediated coupling remains a foundational strategy for linking carboxyl groups on VLPs to primary amines on antigens. In this mechanism, EDC activates carboxyls to form an O-acylisourea intermediate, which is subsequently stabilized by Sulfo-NHS to generate a semi-stable NHS ester amenable to nucleophilic attack by primary amines (Figure 2A; Park et al., 2023). This method has been successfully deployed to display the SARS-CoV-2 RBD on tobacco mosaic VLPs (Royal et al., 2021) and *Burkholderia* Hcp1 on P22 VLPs (at pH 5.5) (Khakhum et al., 2024), demonstrating its utility across diverse capsid geometries.

NaIO_4_-mediated oxidative amine coupling: for aryl amines, periodate oxidation enables a rapid chemoselective coupling mechanism with N, N-dialkyl-N′-acyl-p-phenylenediamines, allowing for attachment under mild aqueous conditions (Figure 2B; Hooker et al., 2006; Liu et al., 2006; Tong et al., 2009). This strategy has been specifically applied to conjugate fibrin or peptide antigens to MS2 VLPs (Obermeyer et al., 2014).

Maleimide-thiol reactions: thiol-selective maleimides enable precise, high-affinity covalent linkage to cysteine residues. Sulfosuccinimidyl-4-(N-maleimidomethyl) cyclohexane-1-carboxylate (Sulfo-SMCC) is frequently used to bridge peptide antigens to cysteine-exposed VLP surfaces at physiological pH (6.5–7.5) (Figure 2C; Ikwuagwu and Tullman-Ercek, 2022; Park et al., 2023). Representative applications include the display of M2E influenza peptides on CCMV cysteine mutants (Cantin et al., 2011), OVA attachment to sHsp (Richert et al., 2012), and the encapsulation or surface-packaging of HAP-GFP in HBV VLPs (Hussain et al., 2024). Advanced refinements, such as the MS2 VLP T15C mutant, have utilized this chemistry to create the first generation of fluorescently-labeled VLP nanoparticles (Peabody, 2003; Pumpens et al., 2016).

Heterobifunctional crosslinkers: heterobifunctional linkers, such as SMPH or SM(PEG)_4_, combine amine-reactive NHS-esters and thiol-reactive maleimide groups at opposing termini, forming stable covalent bridges (Figure 2D; Bárcena and Blanco, 2013). This has enabled the presentation of TRIO epitopes on Qβ VLPs (Francian et al., 2025), and small peptides on RHDV VLPs, enhancing T-helper cell engagement by 700-fold (Peacey et al., 2007). Similar strategies support the presentation of SARS-CoV-2 S proteins (Zha et al., 2021), LCMV P33 peptides (Mohsen et al., 2017a), and Dengue virus (DENV) epitopes (Warner and Frietze, 2021).

Rational residue engineering and sulfhydryl conversion: when native cysteines are absent or poorly accessible, genetic introduction of cysteine (e.g., CCMV R82C/S130C; HEV N573C) or chemical conversion of lysines via Traut’s reagent (2-iminothiolane) provides the necessary sulfhydryl handles for site-specific conjugation (Chen et al., 2018; Jue et al., 1978; Zepeda-Cervantes et al., 2020). This engineered site-specificity represents a transition from stochastic labeling toward a more “programmable” chemical architecture.

##### 2.1.3.2 Advanced precision modification: bio-orthogonal click chemistry and site-specific ligation

Tyrosine-targeted alkyne-azide cycloaddition (CuAAC): tyrosine residues can be selectively modified via diazonium chemistry (pH 9, 4 °C) to introduce terminal alkynes, which then undergo copper(I)-catalyzed azide-alkyne cycloaddition (CuAAC) to generate 1,4-disubstituted 1,2,3-triazole linkages (Figure 2E; Bruckman et al., 2008; Kaur et al., 2021; Patel and Swartz, 2011; Pumpens et al., 2016). This bio-orthogonal strategy supports the attachment of antibody fragments, GM-CSF, nucleic acids, and PEG to VLPs (Patel and Swartz, 2011; Shirbaghaee and Bolhassani, 2016), as well as *Chlamydia* antigens on HepB core SS1 VLPs (Chan, 2014). The incorporation of methionine analogs with alkyne side chains (e.g., azido-propyl-glycine) further extends this toolbox, enabling post-translational decoration with high spatial control (Pumpens et al., 2016).

Orientation-defined functionalization via N-terminal proline coupling: the unique secondary amine of N-terminal prolines provides a distinct site for oxidative coupling. Phenols are oxidized into ortho-quinone intermediates [via K_3_Fe(CN)_6_ or NaIO_4_], which react specifically with prolines to form covalent adducts (Figure 2F; Maza et al., 2020). Brauer et al. (2019) utilized this strategy to achieve highly site-specific conjugation on MS2 VLPs, illustrating a rare example where chemical methods achieve orientation-defined functionalization comparable to enzymatic ligation.

##### 2.1.3.3 Strategic considerations and outlook: toward chemo-enzymatic hybrids

Chemical conjugation remains an essential pillar of the VLP engineering toolkit due to its cargo-agnostic flexibility and post-assembly modularity. It is particularly indispensable for early-stage antigen screening, the presentation of non-proteinaceous targets (glycans/lipids), and as a robust fallback when biological expression fails. Moving forward, the field is evolving toward “chemo-enzymatic” hybrids and strain-promoted azide-alkyne cycloaddition (SPAAC), which aim to reconcile the broad compatibility of chemistry with the positional precision of biological systems. These refinements ensure that chemical strategies will continue to drive the development of multi-antigen co-display platforms and precision-engineered vaccine candidates.

##### 2.1.3.4 Decision triggers for chemical conjugation selection

Chemical conjugation becomes the primary engineering option when the cargo is non-proteinaceous (e.g., synthetic glycans, lipids, nucleic acids) or when genetic fusion/modular assembly fails to yield structurally stable particles.

It is particularly suited for early-stage antigen screening or for applications prioritizing maximal cargo flexibility over strict stoichiometric uniformity.

Transition to genetically encoded systems is required when vaccine efficacy critically depends on fixed inter-antigen spacing (10–30 nm) for optimal B cell receptor cross-linking, or when stochastic labeling introduces unacceptable batch variability for regulatory translation.

#### 2.1.4 Strategic synthesis: a decision-making framework for VLP functionalization

The selection of an antigen presentation strategy constitutes a multidimensional engineering decision rather than a purely technical preference. It requires balancing thermodynamic compatibility, geometric determinism, cargo complexity, immunological precision, and manufacturability. To operationalize these variables, we propose a logic-based decision matrix (Table 1) that aligns platform selection with the specific biophysical and translational requirements of the vaccine candidate.

##### 2.1.4.1 The engineering continuum: coupling constraint to functional agnosticism

Virus-like particle functionalization strategies can be conceptualized along a continuum defined by the degree of thermodynamic coupling between scaffold assembly and antigen incorporation:

- Genetic fusion (the fully coupled regime): this remains the most structurally deterministic strategy, ensuring strict 1:1 stoichiometry and genetic stability. Because antigen incorporation is synchronized with capsid translation and folding, epitope density is inherently encoded within the scaffold architecture. This is the preferred approach for small, structurally simple peptides (typically ≤ 50 aa) where symmetry preservation is paramount (Section “2.1.1 Genetic fusion: balancing antigen presentation with structural integrity”).
- Modular strategies (the programmed decoupled regime): systems such as SpyTag/SpyCatcher and the Streptavidin–Biotin platform exemplify programmed thermodynamic decoupling. Scaffold assembly and antigen folding occur independently, followed by post-assembly covalent or high-affinity docking. This architecture reconciles structural autonomy with geometric determinism, making it ideal for high-value recombinant antigens (e.g., viral trimers or RBDs) where conformational epitope exposure is critical (Section “2.1.2 Modular strategies: interaction-programmed “plug-and-display” architectures”).
- Chemical conjugation (the agnostic regime): chemical strategies represent the most functionally agnostic domain. By fully decoupling scaffold biogenesis from antigen attachment, they enable conjugation of proteins, glycans, lipids, and synthetic molecules. The engineering trade-off is intrinsic stochasticity, necessitating site-specific chemistries to preserve the optimal 10–30 nm inter-antigen spacing required for robust BCR cross-linking (Section “2.1.3 Platform strategy: chemical conjugation–maximum decoupling and cargo flexibility”).

##### 2.1.4.2 Decision-guided selection criteria: a hierarchy of constraints

To implement this framework, we propose a hierarchy centered on three primary engineering determinants:

- Structural complexity and PTM thresholds: cargoes exceeding ∼30–50 kDa, containing complex disulfide networks, or requiring eukaryotic post-translational modifications (PTMs) should pivot away from genetic fusion toward modular or chemical approaches to prevent catastrophic perturbation of the capsid assembly energy landscape.
- Epitope orientation sensitivity: if protective efficacy depends on conformationally constrained or spatially directional epitopes, orientation-defined systems (e.g., enzymatic ligation or SpyTag-mediated docking) are mandatory. Stochastic chemical labeling should be avoided in these cases to prevent epitope masking and ensure high-affinity antibody maturation.
- Manufacturing architecture: genetic fusion offers a streamlined, single-stream production pipeline, minimizing batch variability. In contrast, modular and chemical strategies necessitate dual-stream manufacturing and precisely calibrated conjugation workflows to ensure the regulatory robustness and batch-to-batch reproducibility required for clinical translation.

##### 2.1.4.3 Strategic outlook: hybrid architectures and programmable VLP systems

The next evolutionary stage of VLP engineering lies in hybrid architectures, in which multiple presentation modalities are integrated on a single scaffold to achieve functional synergy. For example, a platform may employ genetic fusion for internal nucleic acid encapsulation (Section “2.2 Intra-VLP nucleic acid encapsulation technologies”), while simultaneously using SpyTag-mediated display for external recombinant antigens. Such combinatorial configurations enable multi-antigen co-display and spatial compartmentalization of adjuvants, underpinning the emergence of “smart” VLP vaccines–engineered nanostructures capable of precise antigen density control and adaptive immune programming.

### 2.2 Intra-VLP nucleic acid encapsulation technologies

While Section “2.1 Extra-VLP protein antigen display technologies” detailed the spatial orchestration of surface antigens to dictate immune recognition, the interior lumen of the VLP offers a sequestered and shielded microenvironment for the encapsulation of functional nucleic acids, such as immunostimulatory CpG oligonucleotides or therapeutic mRNA. Together, surface display and internal cargo loading constitute the two synergistic pillars of VLP engineering, enabling the development of integrated “all-in-one” platforms that synchronize precise antigenic signaling with programmed molecular instructions.

In this section, we examine a continuum of encapsulation strategies–ranging from spontaneous electrostatic co-assembly to highly programmable, sequence- and structure-guided packaging. Each approach is evaluated through our established decision-making framework, weighing the trade-offs between cargo flexibility, loading specificity, functional efficiency, and manufacturability to provide a roadmap for the rational design of multifunctional VLP therapeutics.

#### 2.2.1 Disassembly–reassembly: physical entrapment in the post-assembly regime

**FIGURE 3:**
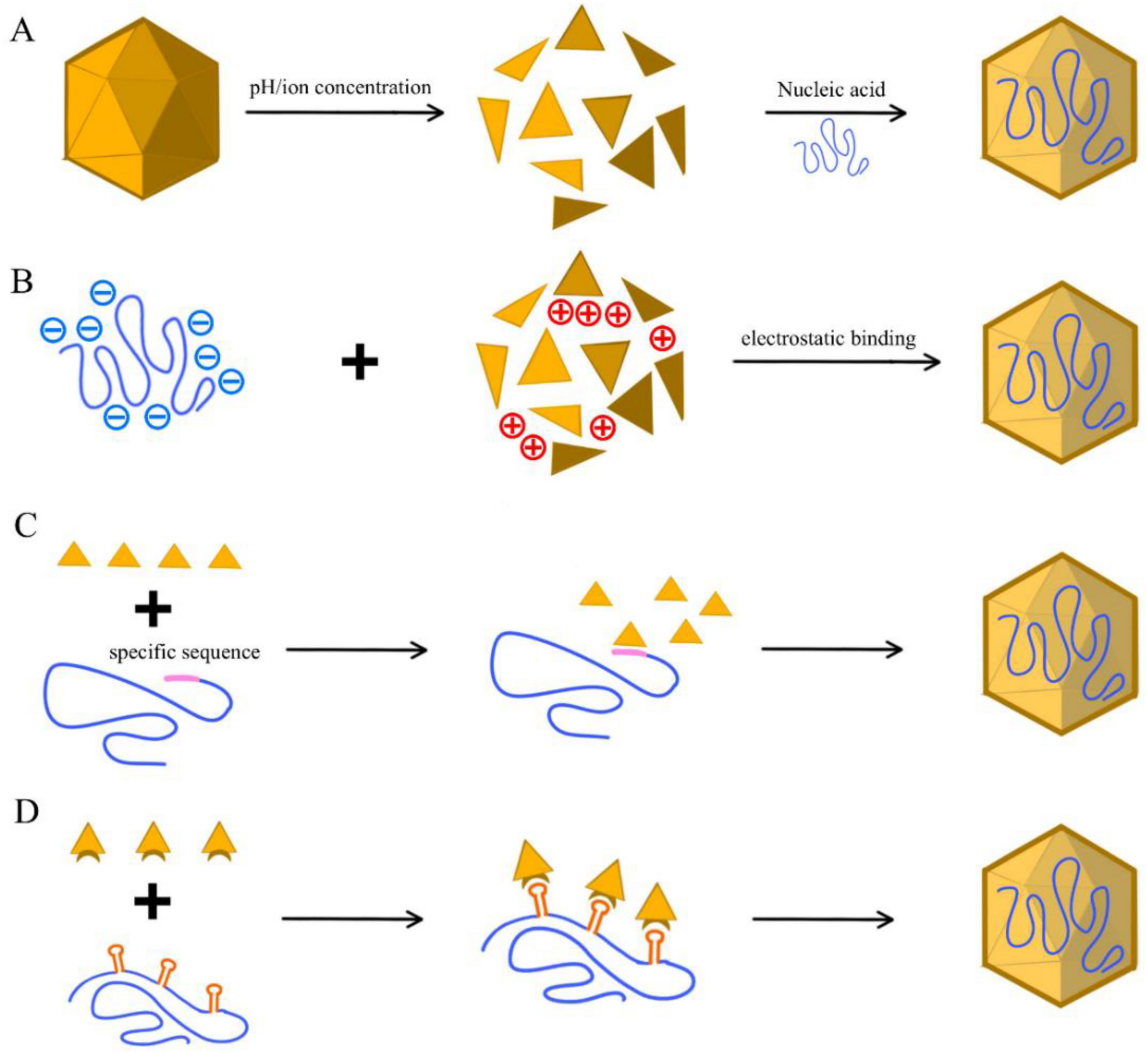
Engineering modalities for intra-VLP nucleic acid encapsulation. **(A)** Disassembly–reassembly: physical entrapment of nucleic acids through the reversible dissociation and restoration of VLP quaternary structures, typically mediated by the titration of redox and chelating agents. **(B)** Electrostatic co-assembly: charge-driven encapsulation within the VLP interior via non-specific interactions between cationic domains of structural proteins and negatively charged nucleic acid phosphate backbones. **(C)** Sequence-specific packaging: targeted recruitment of RNA cargo facilitated by high-affinity binding between specialized capsid protein motifs and defined viral packaging signals (e.g., stem-loop structures). **(D)** RNA aptamer–ABP interaction: Programmed loading mediated by the selective recognition between an RNA-aptamer tag and its cognate aptamer-binding protein (ABP) fused to the VLP inner surface.

The disassembly–reassembly strategy represents a classic post-assembly, physical approach to VLP functionalization. This technique leverages the reversible self-assembly properties of viral capsid proteins, which can be dismantled under controlled environmental stressors–such as high salt concentrations, chelators, or reducing agents–and subsequently triggered to reform in the presence of target cargo (Figure 3A; Balke and Zeltins, 2019; Teunissen et al., 2013). Because this process occurs after the primary biogenesis of the capsid and does not necessitate genetic modification of the structural proteins, it effectively bypasses the expression and folding hurdles often associated with traditional genetic fusion. Consequently, capsid proteins for these platforms can be produced in high-yield microbial hosts, optimized to balance manufacturing cost with assembly fidelity.

The inherent mechanism of bulk physical entrapment, rather than specific molecular recognition, allows this strategy to accommodate an exceptionally diverse repertoire of cargoes, including plasmid DNA, mRNA, siRNA, and even non-nucleic acid small molecules (Beyer et al., 2001; Kim and Kim, 2017; Pechsrichuang et al., 2021). For instance, HPV L1 VLPs have been successfully utilized to encapsulate plasmids encoding reporter genes (GFP, β-gal) or respiratory syncytial virus (RSV) antigens, confirming the functional integrity of the encapsulated material (Buck and Thompson, 2007; Graham et al., 2010; Shirbaghaee and Bolhassani, 2016). However, this adaptability is fundamentally constrained by the finite internal capsid volume and its associated thermodynamic closure energy barrier. As demonstrated by Murine polyomavirus VP1 VLPs under osmotic shock, encapsulation efficiency is highly size-dependent; nucleic acids exceeding 3 kb often face steric exclusion, leading to incomplete capsid closure or structural malformation (Forstová et al., 1995; Tegerstedt et al., 2005).

Despite this versatility, the lack of a defined molecular recruitment logic renders the packaging process inherently stochastic, leading to significant fluctuations in loading efficiency and a heterogeneous product population consisting of fully loaded particles, empty shells, and unintended surface-adsorbed cargo. These challenges extend to the manufacturing regime, which requires precise thermodynamic titration of ionic strength, dialysis gradients, and redox potentials to prevent irreversible protein aggregation or misfolding (Kim and Kim, 2017; Teunissen et al., 2013). Unlike genetically encoded assembly processes that proceed under cellular homeostasis, this regime depends on precise *in vitro* environmental control. While optimized protocols–such as the urea-assisted disassembly of AP205 VLPs–have achieved RNA incorporation rates exceeding 90% while maintaining structural integrity (Wong and Yang, 2025), the extreme sensitivity of these workflows remains a primary bottleneck for high-throughput industrial scaling and batch-to-batch consistency.

##### 2.2.1.1 Decision triggers for disassembly–reassembly selection

The primary utility of this strategy lies in early-stage exploratory research where maximum cargo versatility and proof-of-principle functional delivery are prioritized over immediate scalability or rigorous product homogeneity. However, as projects transition toward advanced translational applications, the inherent stochasticity of physical entrapment and the strict 3 kb thermodynamic limit for cargo size often necessitate a shift toward more programmable, sequence-guided encapsulation strategies to ensure the stoichiometric occupancy and structural stability required for clinical-grade manufacturing.

#### 2.2.2 Charge-mediated strategy: electrostatic encapsulation

Charge-mediated, or electrostatic, encapsulation is a minimal-engineering strategy that leverages the intrinsic or engineered charge landscape of a viral capsid to sequester oppositely charged nucleic acids (Figure 3B). This mechanism parallels the early packaging events observed in many natural viruses, relying on non-covalent, charge-driven associations to load DNA or RNA either through direct adsorption onto cationic interior surfaces or via cationic polymer bridging strategies, such as the use of polyethyleneimine (PEI) (Cadena-Nava et al., 2012; Hill et al., 2018; Rohovie et al., 2017; Shirbaghaee and Bolhassani, 2016; Teunissen et al., 2013). In this regime, VLP scaffolds–commonly sourced from bacterial or insect expression systems–are selected primarily based on the intersection of capsid stability and the density of their internal charge distribution. In contrast to sequence-guided recruitment mechanisms, this approach lacks an encoded selectivity filter and therefore operates purely under physicochemical equilibrium constraints (Cadena-Nava et al., 2012; Garmann et al., 2016; Zlotnick, 2003).

The primary engineering advantage of this strategy lies in its operational agility and manufacturability. Unlike more complex genetic or structural programming, cargo loading is often reduced to controlled mixing under mild buffer conditions, effectively bypassing the need for covalent modification, complex genetic engineering, or multi-step assembly cycles (Sun et al., 2024b). This simplicity positions electrostatic sequestration as an ideal tool for rapid prototyping and high-throughput screening of a broad repertoire of nucleic acids, including mRNA, siRNA, and plasmid DNA (Hill et al., 2018; Shirbaghaee and Bolhassani, 2016). Representative applications across diverse platforms illustrate this utility: the HBV core particle (HBc) naturally utilizes its arginine-rich C-terminal domain (ARD) to engage nucleic acids and regulate assembly, providing a native template for charge-mediated packaging (Rohovie et al., 2017). Similarly, CCMV, P22, and Qβ VLPs have been extensively employed to complex nucleic acids for vaccine development by leveraging their positively charged interior surfaces (Bachmann and Jennings, 2010; Comas-Garcia et al., 2012; Douglas and Young, 2006; Fiedler et al., 2010; O’Neil et al., 2011).

However, the convenience of electrostatic loading is counterbalanced by significant constraints in Loading Specificity and Stability. Because the process is governed by bulk charge complementarity rather than precise molecular recognition, specificity remains intrinsically low; competing anionic biomolecules from the host environment or production media can readily co-adsorb, leading to high product heterogeneity. Furthermore, because electrostatic sequestration does not actively coordinate capsid closure, loading efficiency tends to decrease significantly with increasing cargo length or structural rigidity. The capsid lacks the molecular cues to direct productive assembly pathways and overcome the elastic and entropic penalties inherent in encapsulating large, stiff polymers. This reliance on non-covalent interactions also compromises *in vivo* robustness, as the resulting complexes are particularly sensitive to modest increases in ionic strength (e.g., approaching or exceeding physiological ∼150 mM NaCl) or pH fluctuations, which can trigger premature cargo release during downstream purification (Sasaki and Hilvert, 2019; Schwarz et al., 2017). While cationic polymers like PEI can enhance loading, they introduce well-documented cytotoxicity constraints, forcing an explicit engineering trade-off between delivery potency and biological safety (Shirbaghaee and Bolhassani, 2016).

##### 2.2.2.1 Decision triggers for charge-mediated selection

Electrostatic encapsulation is best positioned as an entry-level loading solution–highly accessible and ideal for rapid screening during early-stage vaccine prototyping. However, once project requirements extend to defined cargo stoichiometry, regulatory-grade batch reproducibility (including validated release profiles), or structural integrity under physiological ionic conditions, electrostatic sequestration alone becomes insufficient. At this strategic transition point, the transition toward sequence-guided or structurally programmable encapsulation strategies is not merely advantageous but strategically necessary to ensure the precision and safety required for advanced therapeutic applications.

#### 2.2.3 Programmable packaging strategy: sequence-guided encapsulation

Sequence-guided encapsulation marks a transition from probabilistic entrapment to deterministic molecular programming, exploiting high-affinity, sequence-specific interactions between viral capsid proteins and defined RNA packaging signals (PS) (Figure 3C; Oppenheim et al., 1986; Tomo et al., 2013; Wang et al., 2003). This biomimetic regime often relies on structurally defined RNA stem-loop motifs recognized by basic domains or zinc-finger modules within the capsid interior. By genetically appending a specific PS to the target RNA, the chimeric transcript is preferentially recruited during intracellular assembly, effectively creating a molecular selectivity filter that prioritizes target cargo over the dense background of host cellular transcripts (Segel et al., 2021). Because this strategy leverages native genome-recognition machinery, it preserves the native capsid architecture more effectively than artificial charge-based loading methods.

The principal engineering upgrade of this strategy lies in its superior specificity and stoichiometric occupancy. The PS–capsid interaction, typically operating in the nanomolar affinity range, ensures precise cargo enrichment. Mechanistically, high-affinity PS recognition increases the probability of cooperative nucleation, which in turn promotes rapid capsid closure. This efficient sequestration minimizes nuclease exposure and extends the intracellular expression longevity of the delivered mRNA (Ohtsuka et al., 2023; Unti and Jaffrey, 2023). These advantages are exemplified by the human retroelement PEG10, which has been re-engineered to package heterologous RNAs–including CRISPR-Cas9 components–for high-efficiency gene editing (Segel et al., 2021). Beyond PEG10, similar principles have been implemented in platforms derived from HEV and HIV, where native RNA-packaging motifs are repurposed to achieve selective genome mimicry and programmable cargo enrichment, supporting applications such as personalized cancer vaccines (Haddrick et al., 1996; Panda et al., 2015; Tang et al., 2024).

However, these precision gains impose a clear engineering trade-off regarding cargo flexibility and manufacturing control. The requirement for specific PS architectures–such as the mandatory structural integrity of both PS termini in PEG10–constrains the RNA design space (Segel et al., 2021). Furthermore, this strategy necessitates a coupled biosynthetic regime, where encapsulation is inextricably tied to the intracellular kinetics of the production host. This coupling reduces process modularity, as loading efficiency becomes sensitive to the metabolic state and relative expression kinetics of capsid proteins and engineered RNA, thereby increasing batch-to-batch variability relative to cell-free post-assembly methods (Segel et al., 2021; Zhou and Routh, 2020).

##### 2.2.3.1 Decision triggers for sequence-guided selection

Sequence-guided encapsulation is the optimal strategic choice when minimizing off-target packaging and ensuring intracellular expression longevity are the primary clinical mandates. While it offers superior selectivity relative to charge-mediated strategies, its adoption requires a higher tolerance for complex RNA architectures and a less modular manufacturing workflow. When cargo design independence from viral motifs and maximal structural modularity become the primary drivers, the strategic transition toward structure-guided methodologies is warranted.

#### 2.2.4 Structure-guided strategy: programmable decoupling via aptamer–ABP pairs

Structure-guided encapsulation represents the most advanced regime of programmable loading, utilizing orthogonal, “lock-and-key” interactions between engineered RNA/DNA aptamers and their cognate binding proteins (ABPs) to direct cargo sequestration (Figure 3D; Balke and Zeltins, 2019; Schwarz et al., 2017). Unlike sequence-guided methods that rely on native viral motifs, this strategy typically employs heterologous pairs–such as the MS2 coat protein (MCP) or PP7 (PCP) systems–where an aptamer hairpin is integrated into the target RNA while the ABP is genetically fused to the VLP structural protein (Gupta et al., 2024; Lyu and Lu, 2022). During intracellular co-expression, the nanomolar-range affinity of aptamer–ABP pairs spatially coordinates cargo recruitment, promoting cooperative nucleation at the site of capsid assembly. This targeted anchoring to the internal capsid surface during early formation accelerates closure, reduces nuclease exposure, and thereby prolongs intracellular stability and protein expression of the payload (Unti and Jaffrey, 2023).

The hallmark of this approach is its maximal modularity and stoichiometric precision. By decoupling cargo recognition from the capsid’s intrinsic sequence requirements, the aptamer–ABP interface functions as a universal adapter, achieving Loading Specificity that is virtually independent of the viral scaffold’s native biology. In applications such as CRISPR-Cas9 sgRNA delivery, this structure-guided recruitment has achieved encapsulation efficiencies exceeding 80%, providing a high degree of Stoichiometric Occupancy that significantly outperforms stochastic entrapment methods (Gupta et al., 2024; Lyu and Lu, 2022). Furthermore, the system allows for sophisticated functional tuning, such as the inclusion of protease-cleavable linkers to control cargo release, reinforcing its status as a highly tunable Cargo Flexibility platform compatible with mRNA, circRNA, and siRNA (Lyu and Lu, 2022; Teunissen et al., 2013; Vaidya and Solomon, 2022).

However, this precision is balanced by substantial Manufacturability challenges. Achieving regulatory-grade batch consistency requires navigating a complex multi-component optimization landscape, maintaining a fragile equilibrium between aptamer folding, ABP-capsid fusion integrity, and the relative expression kinetics of all components within the host cell (Lyu and Lu, 2022; Schwarz et al., 2017). These interdependencies often result in significant batch-to-batch variability and elevated development costs. While established platforms like the MCP-MS2 system have successfully packaged PSA mRNA and circRNA for sustained antigen expression, the sensitivity of the protein-RNA assembly interface remains a primary bottleneck for industrial scale-up, necessitating rigorous, platform-specific empirical tuning (Austin et al., 2002; Gupta et al., 2024; Hattman et al., 1991; Li et al., 2014; Lim et al., 2001; Lu et al., 2021; Lyu and Lu, 2022; Unti and Jaffrey, 2023; Yao et al., 2021).

##### 2.2.4.1 Decision triggers for structure-guided selection

Structure-guided encapsulation is the optimal strategic choice when absolute cargo specificity and modular design independence from viral motifs are the primary design drivers. It is uniquely suited for advanced therapeutics where off-target packaging must be virtually eliminated and where the cargo (e.g., large circRNA or multiplexed sgRNAs) requires active recruitment to ensure viability. However, the associated costs in design complexity and manufacturing sensitivity position it as a specialized, high-performance tool, to be prioritized when precision-driven translational mandates surpass operational simplicity or cost-efficiency considerations.

#### 2.2.5 Strategic synthesis: a decision-making framework for interior loading

The selection of an interior loading strategy constitutes a multidimensional engineering decision rather than a purely technical preference. It requires balancing biomimetic fidelity, stoichiometric determinism, and manufacturing modularity. To operationalize these variables, we propose a logic-based decision matrix (Table 2) that aligns the encapsulation regime with the specific biophysical constraints of the nucleic acid cargo and the translational requirements of the therapeutic target.

##### 2.2.5.1 The engineering continuum: from stochastic entrapment to information-driven programming

Virus-like particle interior loading strategies can be conceptualized along a continuum defined by the degree of molecular recognition between the capsid architecture and the intended cargo:

- Physical and charge-mediated strategies (the bulk-driven regime): these represent the most accessible domains of the continuum, relying on thermodynamic randomness or coulombic affinity. By utilizing disassembly-reassembly or internal charge landscapes, these methods decouple cargo identity from sequence-specific constraints, offering maximal flexibility for rapid prototyping. The engineering trade-off is stochasticity, which inherently limits the “selectivity filter” and poses challenges for regulatory-grade homogeneity (Sections “2.2.1 Disassembly–reassembly: physical entrapment in the post-assembly regime” and “2.2.2 Charge-mediated strategy: electrostatic encapsulation”).
- Sequence-guided strategies (the biomimetic regime): this regime transitions toward deterministic control by exploiting evolutionarily optimized packaging signals (PS). By mirroring natural viral life cycles (e.g., PEG10 or HIV-based systems), these strategies synchronize cargo recruitment with capsid assembly. This is the preferred approach for ensuring high-fidelity loading of target mRNA while minimizing the encapsulation of host-cell transcripts (Section “2.2.3 Programmable packaging strategy: sequence-guided encapsulation”).
- Structure-guided strategies (the orthogonal regime): representing the pinnacle of the continuum, aptamer-ABP systems exemplify programmable thermodynamic decoupling. By utilizing synthetic “lock-and-key” pairs, this architecture achieves absolute cargo specificity independent of the viral scaffold’s native biology. This allows the VLP interior to function as a modular “plug-and-play” compartment for complex payloads like circRNA or multiplexed sgRNAs (Section “2.2.4 Structure-guided strategy: programmable decoupling via aptamer–ABP pairs”).

##### 2.2.5.2 Decision-guided selection criteria: a hierarchy of constraints

To implement this framework, we propose a hierarchy centered on three primary engineering determinants:

- Cargo identity and scale thresholds: for small molecules or high-volume non-specific payloads, physical entrapment remains optimal. However, once cargo length exceeds ∼3 kb or exhibits high structural rigidity, the system should pivot toward sequence- or structure-guided active recruitment to overcome the entropic penalties of encapsulation and ensure native capsid architecture.
- Ionic and environmental sensitivity: if the therapeutic profile requires robust performance in complex biological fluids, electrostatic-based strategies must be scrutinized. Systems relying purely on charge complementarity often face premature dissociation when approaching physiological ∼150 mM NaCl, necessitating a shift toward higher-affinity, sequence-level programming to ensure functional stability.
- Manufacturing architecture and batch reproducibility: while physical mixing and charge-mediated loading offer streamlined post-assembly workflows, programmable strategies (PS or Aptamer-mediated) necessitate coupled biosynthetic regimes. These increases manufacturing overhead, requiring precisely calibrated expression kinetics between the capsid and cargo to mitigate the batch-to-batch variability inherent in multi-component intracellular assembly.

##### 2.2.5.3 Strategic outlook: programmable nanoreactors and adaptive delivery

The next evolutionary stage of VLP interior engineering lies in programmable nanoreactors, in which the internal cavity is not merely a static container but a controlled biochemical environment. By integrating structure-guided loading with stimulus-responsive linkers or co-encapsulated adjuvants, researchers can create “smart” delivery systems capable of adaptive cargo release. This synergy between internal programming (Section “2.2 Intra-VLP nucleic acid encapsulation technologies”) and external functionalization (Section “2.1 Extra-VLP protein antigen display technologies”) marks the transition from simple carrier particles to sophisticated, multi-functional therapeutic nanomachines.

## 3 Translational decision framework for VLP vaccine engineering

**FIGURE 4:**
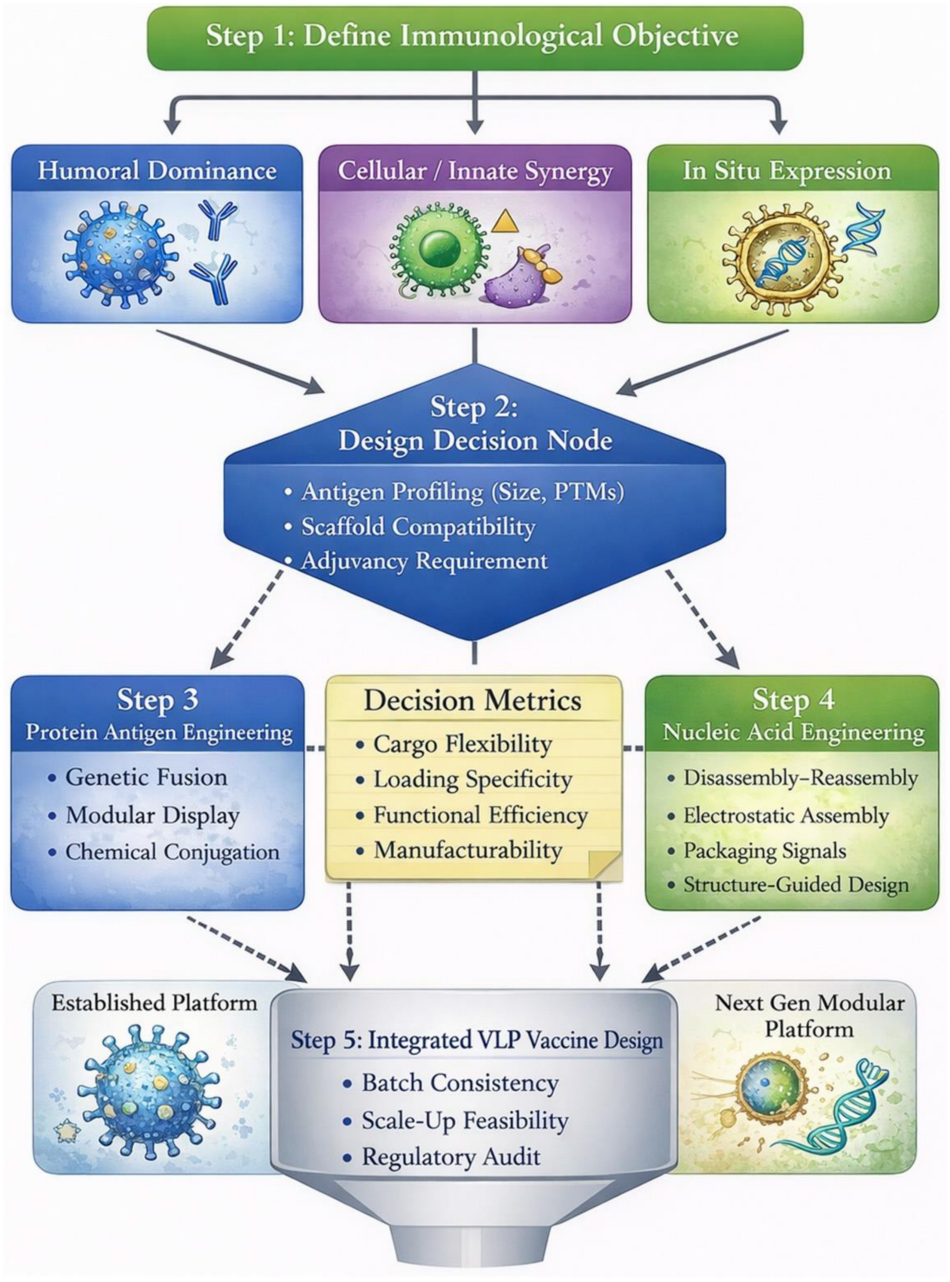
Integrated translational decision framework for rational VLP vaccine engineering. This framework organizes diverse VLP engineering strategies into a structured five-step design logic that links technological selection with specific vaccine objectives. Step 1: Define Immunological Objective. Vaccine design begins by identifying the primary immunological goal, including induction of humoral antibody responses, activation of cellular and innate immune pathways, or in situ antigen expression. Step 2: Design Decision Node. Antigen characteristics (e.g., molecular size and post-translational modification requirements) together with scaffold compatibility are evaluated to guide the selection of an appropriate engineering pathway. Steps 3 and 4: Dual-Stream Engineering. The design pathway diverges into two parallel streams: exterior protein antigen engineering (genetic fusion, modular display strategies, and chemical conjugation) and interior nucleic acid engineering (disassembly–reassembly, electrostatic assembly, and packaging signal–guided encapsulation). These approaches are assessed using four key decision metrics: cargo flexibility, loading specificity, functional efficiency, and manufacturability. Step 5: Translational and Manufacturing Audit. Engineering components converge in an integrative evaluation that considers batch consistency, scalability, and regulatory feasibility to establish an optimized VLP vaccine configuration. The framework concludes at the stage of engineering integration to emphasize rational design rather than downstream biological outcomes.

The four evaluative dimensions introduced in Sections “2.1 Extra-VLP protein antigen display technologies” and “2.2 Intra-VLP nucleic acid encapsulation technologies”–cargo flexibility, loading specificity, functional efficiency, and manufacturability–converge into a translational design logic integrating antigen display and nucleic acid encapsulation within a unified development pathway for rational VLP vaccine engineering (Figure 4).

### 3.1 Step 1–define the immunological objective

Neutralization-focused designs: dense, repetitive antigen arrays on VLPs enhance B cell receptor cross-linking and drive robust neutralizing antibody responses (Laliberte et al., 2025); exterior multivalent display is therefore prioritized when neutralization represents the dominant protective mechanism.

T cell–augmented or PRR-engaging designs: encapsulated immunostimulatory nucleic acids activate antigen-presenting cells via endosomal pattern recognition receptors such as TLR9 (Storni et al., 2004), reinforcing T cell priming alongside surface antigen presentation.

*In situ* antigen expression: VLP platforms capable of RNA packaging enable intracellular antigen production, shifting design strategies toward integrated “display-and-delivery” architectures (Comas-Garcia et al., 2017; Yang et al., 2025; Yin et al., 2025).

### 3.2 Step 2–assess antigen structural compatibility

Size and complexity: direct genetic fusion of structurally complex or conformationally sensitive antigens has been shown to compromise capsid assembly or particle stability. Modular coupling strategies have accordingly been adopted to preserve assembly competence while expanding antigen compatibility (Brune and Howarth, 2018); empirical validation is therefore preferred over rigid molecular thresholds.

Post-translational modifications: host expression systems determine post-translational processing (e.g., glycosylation) required for correct folding and epitope preservation. Expression platform selection (insect versus mammalian) thus constrains scaffold choice and presentation authenticity (Raji et al., 2024).

Orientation sensitivity: site-specific coupling systems (e.g., SpyTag/SpyCatcher, sortase-mediated ligation) enable defined antigen orientation and reduce heterogeneity relative to stochastic chemical conjugation approaches (Antos et al., 2017; Zakeri et al., 2012).

### 3.3 Step 3–select surface display strategy

Genetic fusion: genetic fusion minimizes manufacturing complexity and underlies licensed VLP vaccines (e.g., HBsAg and HPV L1), offering intrinsic multivalent display within a single-component construct.

Modular “plug-and-display”: peptide tagging platforms support flexible multivalent or mosaic antigen presentation and are well suited for multi-epitope or heterologous antigen assemblies (Fougeroux et al., 2025).

Chemical conjugation (the agnostic regime): for non-proteinaceous cargoes (e.g., glycans, lipids) or rapid prototyping of complex antigens, chemical conjugation offers maximum functional flexibility. While this approach decouples scaffold biogenesis from antigen attachment, it necessitates site-specific chemistries to mitigate stochastic distribution and ensure the precise inter-antigen spacing required for optimal B cell activation.

Experimental validation across modalities: regardless of the chosen strategy, experimental verification of particle integrity and functional antigen exposure remains essential. This step serves as a key feedback loop in the rational design workflow, ensuring that the selected engineering approach achieves the intended structural and immunological outcomes.

### 3.4 Step 4–evaluate interior cargo integration

To align with the Engineering Continuum (Table 2), the framework evaluates four distinct interior pathways:

Physical entrapment (post-assembly): reserved for exploratory research requiring maximum cargo versatility (including small molecules or enzymes) via disassembly–reassembly cycles (Fang et al., 2018; He et al., 2022).

Bulk-driven/electrostatic: optimal for rapid screening of small anionic cargoes, provided they can withstand physiological ionic strengths (∼150 mM NaCl) without premature dissociation (He et al., 2022).

Sequence-guided (biomimetic): recommended when minimizing host-cell transcript contamination is critical. This path utilizes native Packaging Signals (PS) to achieve high-fidelity enrichment (e.g., PEG10 or HIV-based systems) (Raguram et al., 2022; Stockley et al., 2013).

Structure-guided (orthogonal): the “pinnacle” of precision, employing Aptamer-ABP pairs (e.g., MS2-MCP) for absolute stoichiometric control of complex payloads like circRNA or multiplexed sgRNAs (Lyu et al., 2020; Raguram et al., 2022).

### 3.5 Step 5–translational and manufacturing considerations

Reproducibility and complexity: multi-component assemblies introduce additional quality control variables relative to single-component constructs, influencing batch consistency and scale-up feasibility (Earnest et al., 2026).

Regulatory familiarity: clinically validated backbones with established safety and manufacturing precedents reduce regulatory uncertainty. HBsAg and HPV L1 platforms provide well-documented licensure pathways.

### 3.6 Concluding perspective

This framework supports sequential evaluation of immunological objectives, structural compatibility, surface engineering modality, interior programming requirements, and translational constraints. By aligning VLP configurations with biological function and manufacturing feasibility, it advances rational vaccine design without imposing rigid thresholds.

## 4 VLP-based vaccines for infectious diseases: from engineering design to clinical reality

The clinical translation of VLP technology has transitioned from the empirical replication of natural viral structures toward the deployment of highly engineered, programmable scaffolds. This section evaluates how engineering strategies–ranging from native co-expression to modular coupling–have been operationalized across major infectious disease landscapes, providing a consolidated overview of their immunological outcomes and manufacturing trajectories (Table 3). Beyond the primary targets discussed below, VLP candidates for *Mycobacterium tuberculosis* (Dhanasooraj et al., 2016; Wang et al., 2023), dengue fever virus (Parra-González et al., 2025), monkeypox virus (Belghith et al., 2025), rotavirus (Latifi et al., 2023), and Zika virus (Rothen et al., 2024) are currently under active development.

**TABLE 3:** Comprehensive summary of engineering technologies for VLP-based vaccines.

Pathogen category	Targeted pathogens	Engineering technologies
Viral pathogens	Hepatitis B virus (HBV)	Genetic fusion (Lehky et al., 2024; Whitacre et al., 2020; Skrastina et al., 2008; Gedvilaite et al., 2000)
	Hepatitis C virus (HCV)	Genetic fusion (Sominskaya et al., 2010; Masavuli et al., 2017)
	Hepatitis E virus (HEV)	Genetic fusion (Zhu et al., 2010; Cao et al., 2023; Go et al., 2021; Bai et al., 2021)
	Human papillomaviruses (HPV)	Genetic fusion (Tumban et al., 2011, 2015); chemical techniques (Tyler et al., 2014); coiled-coil (Spagnoli et al., 2017); disassembly and reassembly (Mach et al., 2006)
	Human immunodeficiency virus (HIV)	Genetic fusion (Tohidi et al., 2017; Benen et al., 2014; Mogus et al., 2020; Karch et al., 2019; Pastori et al., 2012); chemical techniques (García-Trujillo et al., 2025); SpyTag–SpyCatcher (Peyret et al., 2020; Malebo et al., 2024; Ximba et al., 2022); coiled-coil (Boato et al., 2007; Karch et al., 2019); RNA aptamer-ABP interaction (Sun et al., 2011); specific sequence interaction (Legendre and Fastrez, 2005)
	Influenza virus	Genetic fusion (Vasyagin et al., 2024); SpyTag–SpyCatcher (Heinimäki et al., 2022; Lampinen et al., 2023; Sharma et al., 2020; Thornlow Lamson et al., 2025); sortase-mediated ligation (Patterson et al., 2017); chemical techniques (Jegerlehner et al., 2002; Liu et al., 2020)
	Respiratory syncytial virus (RSV)	Genetic fusion (Quan et al., 2011); disassembly and reassembly (Shirbaghaee and Bolhassani, 2016; Buck and Thompson, 2007; Graham et al., 2010); RNA aptamer-ABP interaction (Gupta et al., 2024)
	SARS-CoV-2/SARS-CoV	Genetic fusion (Sazegari et al., 2023; Azhar et al., 2021; Liu et al., 2021; Hennrich et al., 2021); streptavidin-biotin (Setyo Utomo et al., 2023); sortase-mediated (Olivera-Ugarte et al., 2022); SpyTag–SpyCatcher (Yan et al., 2023; Tan et al., 2021); coiled-coil (Pimentel et al., 2009); RNA aptamer-ABP (Unti and Jaffrey, 2023)
	Zika virus	Genetic fusion (Rothen et al., 2024; Yang et al., 2017; Boigard et al., 2017); chemical techniques (Côrtes et al., 2025; Cabral-Miranda et al., 2019), SpyTag–SpyCatcher (Jung et al., 2025);
	West Nile virus	Genetic fusion (He et al., 2021); SpyTag–SpyCatcher (Stander et al., 2021); chemical techniques (Spohn et al., 2010)
	Mpox virus	SpyTag–SpyCatcher (Belghith et al., 2025)
	Norovirus	Genetic fusion (Wang et al., 2013; Warren et al., 2025; Parra et al., 2012; Tsurumi et al., 2026)
	Enterovirus 71 (EV71)	Genetic fusion (Wang et al., 2021; Ye et al., 2014); sortase-mediated interactions (Tang et al., 2016)
	Emerging viruses	Genetic fusion [Chikungunya (Hamer et al., 2025), Dengue (Suphatrakul et al., 2015), Ebola (Ohimain, 2016), Poliovirus (Sherry et al., 2020), Rotavirus (Vieira et al., 2005)]
Bacterial pathogens	*Mycobacterium tuberculosis*	Genetic fusion (Wang et al., 2023; Dhanasooraj et al., 2016; Krammer et al., 2010; Yin et al., 2011)
	*Shigella*	SpyTag–SpyCatcher mediated interaction (Li et al., 2022)
	*Streptococcus pneumoniae*	Coiled-coil mediated interaction (Tamborrini et al., 2015)
	*Bacillus anthracis*	Genetic fusion (Yin et al., 2014)
	*Borrelia burgdorferi*	Genetic fusion (Marcinkiewicz et al., 2018)
Parasitic pathogens	*Plasmodium* (Malaria)	Genetic fusion (Francica et al., 2021); streptavidin-biotin (Thrane et al., 2015); SpyTag–SpyCatcher (King et al., 2024; Boonyakida et al., 2024; Janitzek et al., 2016); coiled-coil (Karch et al., 2017)
	*Toxoplasma gondii*	Genetic fusion (Eom et al., 2023); coiled-coil (El Bissati et al., 2014)

### 4.1 Hepatitis B, C, and E viruses

Hepatitis B virus vaccines represent the inaugural success of VLP technology and illustrate the transition from single-antigen mimicry toward expanded antigenic breadth. While second-generation vaccines like Engerix-B^®^ and Recombivax HB^®^ rely on the HBsAg S-protein, the emergence of escape mutations such as G145R exposed the limitations of single-determinant designs (Lehky et al., 2024; Whitacre et al., 2020). Consequently, third-generation vaccines like Sci-B-Vac™ utilize genetic fusion to incorporate Middle (M) and Large (L) envelope proteins (Skrastina et al., 2008; Whitacre et al., 2020). By displaying Pre-S1/Pre-S2 epitopes that bind directly to the hepatocyte NTCP receptor, these VLPs simulate the natural infection process and trigger broader B-cell and T-cell responses, achieving high seroprotective rates even in traditional non-responders (Skrastina et al., 2008). Recent advancements in mammalian-expressed S-HBsAg VLPs have further improved assembly status analysis and anti-HBs serology, ensuring high fidelity of the displayed antigens (Lehky et al., 2024).

Beyond native envelope proteins, HBV platforms, particularly the Hepatitis B core (HBc), have served as versatile scaffolds for modular technologies and heterologous antigen display. Through genetic insertion into surface-exposed regions, HBc has been engineered to carry epitopes from diverse pathogens, including influenza M2 extracellular domains (Kazaks et al., 2017) and SARS-CoV-2 segments (Sazegari et al., 2023). Furthermore, the construction of multivalent HBc VLPs carrying both HBV and HCV epitopes has been evaluated to provide potential cross-protection against co-infections (Sominskaya et al., 2010).

In a parallel trajectory, HCV vaccine candidates are progressing through genetic fusion strategies to elicit broadly neutralizing antibodies against diverse viral strains, leveraging optimized preclinical production models (Masavuli et al., 2017; Sominskaya et al., 2010).

The HEV vaccine Hecolin^®^ utilizes an *E. coli*-expressed truncated ORF2 segment (aa 368–606) to form 27 nm VLPs (Cao et al., 2023; Zhu et al., 2010). While achieving 100% efficacy and safety in healthy adults, challenges from genotype 3 variants highlight the importance of structural fidelity for cross-genotype coverage (Zhu et al., 2010). Recent studies utilizing baculovirus-produced HEV-VLPs in swine and rabbit models demonstrate that these particles provide robust protection against diverse HEV strains (Bai et al., 2021; Go et al., 2021), reinforcing the value of the recombinant truncated ORF2 capsid protein platform in ensuring broad-spectrum immunity (Cao et al., 2023).

### 4.2 Human papillomavirus (HPV)

Human papillomavirus vaccine development exemplifies the mastery of valency expansion and manufacturing standardization. From the quadrivalent Gardasil^®^ to the nine-valent Gardasil^®^9, and the next-generation 14-valent recombinant vaccine (SCT1000), engineering has evolved from basic expression in yeast or insect cells (Kim and Kim, 2017) to advanced disassembly–reassembly techniques (Mach et al., 2006). These techniques involve finely tuning the intracellular microenvironment, such as pH and ionic strength, to ensure proper VLP conformation and stability across diverse L1 genotypes (Bei et al., 2024; Mach et al., 2006). For instance, Cervarix^®^, which utilizes the AS04 adjuvant, has demonstrated long-term protective efficacy for over a decade by activating innate signaling pathways to sustain seropositivity (Mohsen et al., 2017b; Yousefi et al., 2022). The recent preclinical assessment of the 14-valent candidate (Bei et al., 2024) further utilizes validated Luminex immunological assays to ensure precise immunogenicity tracking across multiple strains (Zhang et al., 2023).

Beyond the standard L1-VLP platforms, researchers are increasingly targeting the minor capsid protein L2 to achieve broader cross-genotype protection. To overcome the low natural immunogenicity of L2, innovative genetic fusion and modular approaches have been employed. This includes displaying broadly neutralizing L2 epitopes on bacteriophage platforms (e.g., PP7 VLPs) (Tumban et al., 2011, 2015) or utilizing thioredoxin-based multiepitope nanoparticles (Spagnoli et al., 2017). These strategies, often enhanced by coiled-coil mediated interactions (Spagnoli et al., 2017) or chemical conjugation techniques (Tyler et al., 2014), enable the presentation of consensus L2 epitopes that induce broadly neutralizing antibodies against a wide array of HPV types (Tyler et al., 2014), potentially paving the way for a universal “pan-HPV” vaccine.

### 4.3 Human immunodeficiency virus (HIV)

For HIV, given the inherently low immunogenicity of native HIV Env trimers, engineering pivots toward high-density display and modular coupling to overcome epitope accessibility barriers. While genetic fusion remains foundational for co-expressing Gag and Env proteins (Benen et al., 2014; Tarrés-Freixas et al., 2024; Tohidi et al., 2017), researchers are increasingly adopting modular strategies to present complex epitopes like the membrane-proximal external region (MPER) or the fusion peptide (Mogus et al., 2020; Tohidi et al., 2017). A significant advancement is the development of engineered “MinGag-VLPs,” which achieve superior antigen density by fusing gp41 fragments directly to the Gag scaffold. These high-density particles have demonstrated the ability to induce potent, antibody-dependent functional immune responses in preclinical models (Tarrés-Freixas et al., 2023).

The integration of SpyTag–SpyCatcher systems (Malebo et al., 2024; Peyret et al., 2020; Ximba et al., 2022), chemical techniques (e.g., copper-free click chemistry) (García-Trujillo et al., 2025), and coiled-coil interactions (Boato et al., 2007; Karch et al., 2019) allows for the precise, stoichiometric attachment of Env trimers in their native-like trimeric conformations (Karch et al., 2019). Two-component nanoparticle vaccines constructed via SpyTag-mediated coupling have elicited Tier 2 neutralizing antibodies, underscoring the role of modular assembly in overcoming Env-associated immunogenic constraints (Malebo et al., 2024; Ximba et al., 2022).

Furthermore, RNA aptamer–antigen-binding protein (ABP) interactions enable programmable cargo loading, offering a modular route for interior encapsulation within VLP architectures (Sun et al., 2011). By leveraging expression systems such as Saccharomyces cerevisiae, VLP-derived particles have been engineered to package functional heterologous mRNAs (Legendre and Fastrez, 2005) or specific RNA-vaccine platforms (Sun et al., 2011). This reflects a broader shift toward utilizing VLPs not only as immunogens but as highly customizable molecular delivery vehicles for nucleic acid-based therapies.

### 4.4 *Plasmodium* (malaria)

Virus-like particle-based malaria vaccines focus on density-optimized antigen presentation to block hepatocyte invasion. The first WHO-approved vaccine, RTS,S/AS01 (Mosquirix), fuses CSP NANP repeats and T-cell epitopes to the HBsAg scaffold (Hammershaimb and Berry, 2024; Moorthy and Ballou, 2009). To address the limitations of short-lived protection observed in RTS,S (Agnandji et al., 2012), the next-generation R21 vaccine achieves a near 1:1 stoichiometric ratio of CSP-HBsAg to HBsAg through structural optimization (Collins et al., 2017; Hammershaimb and Berry, 2024). By significantly increasing surface antigen density, R21 elicits high-titer antibodies approaching protective thresholds associated with pre-erythrocytic immunity (Aderinto et al., 2024; Collins et al., 2017). Complementing these HBsAg-based designs, genetic fusion strategies have also been applied to Alphavirus VLPs to present CSP junctional epitopes (Francica et al., 2021). These engineered particles elicit potent antibodies targeting the NPDP/NVDP regions, providing a novel layer of protection by neutralizing sporozoites at a critical conserved site (Francica et al., 2021).

To further refine these architectures, researchers are deploying modular strategies to accommodate complex or multi-stage parasitic antigens. The SpyTag–SpyCatcher system has been instrumental in creating full-length CSP-VLP vaccines with enhanced durability (Janitzek et al., 2016), as well as the preclinical development of stabilized RH5-VLP candidates which induce improved antimalarial antibodies against the blood stage (King et al., 2024). Recent innovations also include the modular display of *P. yoelii* CSP and MSP-1 on Noro VLPs, demonstrating the versatility of non-HBV scaffolds (Boonyakida et al., 2024). Additionally, streptavidin–biotin interactions (Thrane et al., 2015) and coiled-coil scaffolds (Karch et al., 2017) enable the precise orientation of antigens such as VAR2CSA for placental malaria.

Beyond human-targeted prevention, plant-produced transmission-blocking candidates (TBVs) represent a critical strategy for global eradication. Utilizing chimeric Alfalfa mosaic virus platforms in plants, candidates like Pfs25-VLP target gametocyte development (Chichester et al., 2018; Jones et al., 2013). Phase 1 dose-escalation studies have confirmed the safety and immunogenicity of these plant-produced VLPs in healthy adults (Chichester et al., 2018), highlighting their potential to disrupt the malaria life cycle.

### 4.5 Influenza virus and respiratory syncytial virus (RSV)

Influenza VLPs address antigenic drift through three primary streams: genetic fusion of conserved M2e epitopes (Kushnir et al., 2012; Shirbaghaee and Bolhassani, 2016; Vasyagin et al., 2024), native trimeric HA mimicry–a principle foundational to successful recombinant platforms like FluBlok^®^ (Quan et al., 2016) –and further extension into true VLP systems incorporating M1 or Gag scaffolds (Norizwan and Tan, 2024). Specialized modular techniques, such as SpyTag–SpyCatcher technology (Heinimäki et al., 2022; Lampinen et al., 2023; Sharma et al., 2020; Thornlow Lamson et al., 2025), sortase-mediated ligation (Patterson et al., 2017), and coiled-coil couplings (Babapoor et al., 2011), allow for the rapid display of multiple HA subtypes on a single immunogen (Thornlow Lamson et al., 2025), ensuring rapid adaptation to seasonal variants and prolonged antibody durability (Hills and Howarth, 2022). Furthermore, innovative packaging signal-directed loading of circRNA within VLPs represents a dual-function breakthrough as both a vaccine candidate and a delivery vehicle (Gupta et al., 2024).

Similarly, RSV vaccine development centers on stabilizing the fusion (F) glycoprotein in its metastable pre-fusion conformation. Structure-guided engineering, exemplified by the DS-Cav1 mutations, effectively locks the F protein in this antigenically optimal state (Crank et al., 2019; McLellan et al., 2013). Building upon modular design principles applied in respiratory platforms (Chu and Quan, 2023), stabilized pre-F trimers are displayed on nanoparticle or VLP scaffolds. A representative implementation is the genetic fusion of RSV-F to structural scaffolds like HIV-Gag, enabling assembly into enveloped VLPs that significantly amplify neutralizing responses *in vivo* (Quan et al., 2011; Trinité et al., 2024).

To further refine these architectures, researchers deploy disassembly and reassembly strategies (Buck and Thompson, 2007; Graham et al., 2010; Shirbaghaee and Bolhassani, 2016) and RNA aptamer-ABP interactions (Gupta et al., 2024) to optimize cargo loading and interior stability. These scaffold-based display systems, whether achieved via genetic fusion or modular assembly (Gupta et al., 2024; Marcandalli et al., 2019; Shirbaghaee and Bolhassani, 2016), support egg-independent production and facilitate scalable manufacturing for clinical deployment in older adults and pregnant populations (Kampmann et al., 2023; Papi et al., 2023).

### 4.6 SARS-CoV-2 and emerging viral threats

The COVID-19 pandemic accelerated the deployment of modular VLP platforms for rapid response. Beyond traditional genetic fusion (Azhar et al., 2021; Hennrich et al., 2021; Liu et al., 2021; Sazegari et al., 2023), the SpyTag–SpyCatcher system has been pivotal for covalent RBD display, achieving nearly 100% site occupancy (Tan et al., 2021; Yan et al., 2023). Advanced assembly techniques, including streptavidin–biotin interactions (Setyo Utomo et al., 2023), sortase ligation (Olivera-Ugarte et al., 2022), and coiled-coil mediated assembly (Pimentel et al., 2009), have enabled multivalent candidates covering variants like Omicron (Mi et al., 2021, 2024). Furthermore, the platform’s versatility is enhanced by RNA aptamer-ABP interactions for delivering circular mRNA (Unti and Jaffrey, 2023).

This programmable flexibility is being extended to other emerging threats. For Mpox, multiprotein VLP nanoparticles have induced potent protection in primate models (Belghith et al., 2025). In Zika and Dengue research, strategies range from genetic fusion of domain III (Boigard et al., 2017; Rothen et al., 2024; Suphatrakul et al., 2015; Yang et al., 2017) to chemical conjugation (Cabral-Miranda et al., 2019; Côrtes et al., 2025) and SpyTag–SpyCatcher (Jung et al., 2025). Similarly, Norovirus candidates benefit from rational design by gene fusion (Parra et al., 2012; Tsurumi et al., 2026; Wang et al., 2013; Warren et al., 2025) and optimized expression in insect or silkworm systems (Lampinen et al., 2024; Tsurumi et al., 2026). These advancements in Chikungunya (Hamer et al., 2025), Ebola (Ohimain, 2016), Poliovirus (Sherry et al., 2020) and Rotavirus (Vieira et al., 2005) reflect a paradigm shift toward modular engineering principles that improve global vaccine accessibility (Saha et al., 2025).

### 4.7 Bacterial and other pathogenic targets

The VLP platform’s versatility extends to complex bacterial and parasitic targets through precise surface engineering. For *Mycobacterium tuberculosis*, strategies include the genetic fusion of key antigens such as HBHA, MTP, and ESAT-6 onto HBc or influenza-based scaffolds (Dhanasooraj et al., 2016; Krammer et al., 2010; Wang et al., 2023; Yin et al., 2011). Similarly, vaccines for *Toxoplasma gondii* leverage VLP-expressed cyst wall proteins to elicit robust mucosal and systemic immunity (El Bissati et al., 2014; Eom et al., 2023).

To address diverse bacterial pathogens, researchers are deploying modular assembly tools: *Shigella* vaccine candidates have been developed using SpyTag–SpyCatcher mediated orthogonal biosynthesis (Li et al., 2022), while *Streptococcus pneumoniae* targets utilize coiled-coil mediated interactions to display B-cell epitopes from the pneumococcal surface protein A (PspA) (Tamborrini et al., 2015). Additionally, genetic fusion remains a cornerstone for displaying protective epitopes of *Bacillus anthracis* on HBc particles (Yin et al., 2014) and enhancing the efficacy of *Borrelia burgdorferi* (Lyme disease) vaccines by conjugating factor H-binding proteins to VLPs (Marcinkiewicz et al., 2018). Together, these advances illustrate how precise surface engineering, enabled by genetic fusion and modular assembly technologies, transforms VLPs from pathogen-mimicking particles into programmable antigen display platforms adaptable to diverse infectious targets.

## 5 Conclusion and future perspectives

Virus-like particles have emerged as versatile nanoscale platforms capable of supporting antigen display and, in engineered systems, nucleic acid delivery (Valenzuela et al., 1982). Through surface multivalency and interior cargo programming, VLP systems provide opportunities to modulate humoral and cellular immune responses in an antigen- and context-dependent manner (Tan et al., 2021; Mi et al., 2024).

This review outlines a translational design framework that links immunological objectives with defined engineering constraints. By organizing antigen structural properties, display modalities, cargo integration strategies, and manufacturing considerations within a sequential decision logic, it provides a structured approach to guide VLP development beyond purely empirical optimization.

The clinical success of VLP-based vaccines derived from hepatitis B surface antigen and human papillomavirus L1, exemplified by licensed products such as Engerix-B^®^ and Gardasil^®^, underscores the translational viability of structurally defined particulate platforms (Valenzuela et al., 1982; Kirnbauer et al., 1992; Joura et al., 2007). Continued refinement of modular coupling technologies and RNA-packaging strategies has expanded the range of engineering configurations for VLP vaccines under investigation against pathogens such as mpox, malaria, and SARS-CoV-2 (Mi et al., 2024; Belghith et al., 2025; Janitzek et al., 2016).

Moreover, advances in computational protein design and generative modeling are increasingly being integrated into VLP engineering workflows (Watson et al., 2023; Divine et al., 2021). The convergence of structured decision frameworks with predictive computational tools offers the potential for more systematic optimization of structural stability, antigen presentation geometry, and manufacturability, paving the way for next-generation, design-driven VLP vaccine development.
